# Development of a Short Version of MSQOL-54 Using Factor Analysis and Item Response Theory

**DOI:** 10.1371/journal.pone.0153466

**Published:** 2016-04-14

**Authors:** Rosalba Rosato, Silvia Testa, Antonio Bertolotto, Paolo Confalonieri, Francesco Patti, Alessandra Lugaresi, Maria Grazia Grasso, Anna Toscano, Andrea Giordano, Alessandra Solari

**Affiliations:** 1 Department of Psychology, University of Turin, Turin, Italy; 2 Clinical Neurobiology Unit, Regional Referral Multiple Sclerosis Centre (CReSM), University Hospital San Luigi Gonzaga, Orbassano, Italy; 3 Unit of Neuroimmunology, Foundation IRCCS Neurological Institute C. Besta, Milan, Italy; 4 MS Centre, Neurology Clinic, University Hospital Policlinico Vittorio Emanuele, Catania, Italy; 5 Department of Neuroscience, Imaging and Clinical Sciences, G. d'Annunzio University of Chieti-Pescara, Chieti, Italy; 6 Multiple Sclerosis Unit, IRCCS S. Lucia Foundation, Rome, Italy; 7 Unit of Neuroepidemiology, Foundation IRCCS Neurological Institute C. Besta, Milan, Italy; University of Illinois at Chicago College of Medicine, UNITED STATES

## Abstract

**Background:**

The Multiple Sclerosis Quality of Life-54 (MSQOL-54, 52 items grouped in 12 subscales plus two single items) is the most used MS specific health related quality of life inventory.

**Objective:**

To develop a shortened version of the MSQOL-54.

**Methods:**

MSQOL-54 dimensionality and metric properties were investigated by confirmatory factor analysis (CFA) and Rasch modelling (Partial Credit Model, PCM) on MSQOL-54s completed by 473 MS patients. Their mean age was 41 years, 65% were women, and median Expanded Disability Status Scale (EDSS) score was 2.0 (range 0–9.5). Differential item functioning (DIF) was evaluated for gender, age and EDSS. Dimensionality of the resulting short version was assessed by exploratory factor analysis (EFA) and CFA. Cognitive debriefing of the short instrument (vs. the original) was then performed on 12 MS patients.

**Results:**

CFA of MSQOL-54 subscales showed that the data fitted the overall model well. Two subscales (Role Limitations—Physical, Role Limitations—Emotional) did not fit the PCM, and were removed; two other subscales (Health Perceptions, Social Function) did not fit the model, but were retained as single items. Sexual Satisfaction (single-item subscale) was also removed. The resulting MSQOL-29 consisted of 25 items grouped in 7 subscales, plus 4 single items. PCM fit statistics were within the acceptability range for all MSQOL-29 items except one which had significant DIF by age. EFA and CFA indicated adequate fit to the original two-factor (Physical and Mental Health Composites) hypothesis. Cognitive debriefing confirmed that MSQOL-29 was acceptable and had lost no key items.

**Conclusions:**

The proposed MSQOL-29 is 50% shorter than MSQOL-54, yet preserves key quality of life dimensions. Prospective validation on a large, independent MS patient sample is ongoing.

## Introduction

Interest in the Health Related Quality of Life (HRQOL) of people with multiple sclerosis (MS) has been driven by a desire to broaden traditional outcome measures to include those not always evident on clinical examination, but nevertheless important to the patient [1, 2]. HRQOL inventories help clinicians appreciate patient priorities and facilitate physician-patient communication and shared decision making. The first HRQOL instruments specific for people with MS were published in the mid-1990s [3,4]. Of these, the Multiple Sclerosis Quality Of Life-54 (MSQOL-54) gained immediate popularity. Originally developed in US English, it has been translated and culturally adapted into several languages [5–11]. In 1999 we produced the Italian version of MSQOL-54, which is currently the most-used HRQOL inventory in Italy [5].

Important limitations of MSQOL-54 are that it was produced without direct involvement of patients, it requires considerable time to complete [2,12] and scores have to be calculated using an algorithm. A long questionnaire is particularly challenging for patients suffering from fatigue, one of the commonest MS symptoms, who are thus likely to miss replies or refuse to compile the instrument. Furthermore the time spent by clinicians gathering and interpreting HRQOL information as part of the clinical encounter is not reimbursed [2]. Questionnaire length, and complexity of score calculation and interpretation, are recognised as main barriers to the use of HRQOL and other patient-reported inventories in everyday practice [2,13].

The availability of a shorter version that is also implemented as software and immediately (and correctly) produces the scores, is likely to improve usability, interpretability and validity.

We decided to develop a short version of the MSQOL-54, available in electronic, patient self-administered form, with automatic scoring. We aimed to preserve both the conceptual model and the psychometric properties of the original instrument [14]. The project has two phases. In the first, presented here, a short version was produced based on psychometric analysis of a database of compiled MSQOL-54 responses together with feedback from MS/HRQOL professionals and people with MS (cognitive debriefing). Our aim was to work with the original set of items and subscales, retaining those that had the best psychometric characteristics. In the second phase (not part of the present manuscript) the short instrument is being validated in an independent sample of 500 people with MS.

### Participants and Methods

We considered MSQOL-54 data hosted at five Italian centres (University Hospital ‘San Luigi Gonzaga’, Foundation IRCCS Neurological Institute ‘C. Besta’, University of Chieti-Pescara, IRCCS S. Lucia Foundation, and University Hospital of Catania). Database records were only eligible if MS was diagnosed according to McDonald [15] or McDonald revised criteria [16], and patient age, sex, and Expanded Disability Status Scale (EDSS) score [17] were available.

The study protocol was approved by the Ethics Committees of the five centres (University Hospital ‘San Luigi Gonzaga’, Foundation IRCCS Neurological Institute ‘C. Besta’, University of Chieti-Pescara, IRCCS S. Lucia Foundation, and University Hospital of Catania). The centre datasets (always baseline data for trials/longitudinal studies) come from research projects carried out in various Italian centres (see below), in hospital or outpatient settings, that were approved by the centre ethics committees. Patients gave written informed consent to being included in the original projects. Additional consent was not required for this secondary analysis, for which patient privacy and anonymity was guaranteed. The MS patients who participated in cognitive debriefing (see below) provided written informed consent to participate.

### Instrument

The MSQOL-54 comprises the generic Short-Form 36-item (SF-36) instrument [18], plus 18 MS-specific items derived from professional opinion and a literature review [3]. The 54 items are organized into 12 multi-item and two single-item subscales (Table 1). These enquire about HRQOL over the preceding month, except item 2 (Change in Health) which refers to the preceding year. As for SF-36, two composite scores (Physical Health Composite, PHC, and Mental Health Composite, MHC) are derived by combining scores of the relevant subscales [3].

**Table 1:** MSQOL-54 subscales and items.

Subscale	No. of items	Item number
Physical Function	10	3–12
Role Limitations–Physical	4	13–16
Role Limitations–Emotional	3	17–19
Bodily Pain	3	21, 22, 52
Emotional Wellbeing	5	24–26, 28, 30
Energy	5	23, 27, 29, 31, 32
Health Perceptions	5	1, 34–37
Social Function	3	20, 33, 51
Cognitive Function	4	42–45
Health Distress	4	38–41
Sexual Function	4	46–49
Change in Health	1	2
Satisfaction with Sexual Function	1	50
Overall Quality of Life	2	53, 54

MSQOL-54, Multiple Sclerosis Quality of Life-54.

The MSQOL-54 has well documented validity in terms of content, constructs, reliability, discrimination [3,5–11], and responsiveness [19].

### Methodology

As a preliminary analysis, we applied confirmatory factor analysis (CFA) [20] to verify the MSQOL-54 original dimensional structure in our study sample. The approach we used to shorten the MSQOL-54 was based on Goetz et al., [14] and proceeded by applying the partial credit model (PCM), a Rasch model for polytomous items, to each original subscale [21]. Misfitting items were removed one at a time and the model re-estimated. Subscales were preserved if at least three items adequately fitted the PCM. Items pruning was supported by input of MS and HRQOL experts (RR, FP, AG, AS) to preserve important HRQOL dimensions at least as single item. The composite scores of the new instrument (called Physical Health Composite [PHC] and Mental Health Composite [MHC] after the original) were then determined using exploratory factor analysis (EFA) and checked by confirmatory factor analysis (CFA). Finally cognitive debriefing of the new instrument (vs. original) was performed on patients from three Italian MS centres. The method of purposive sampling was used in order to obtain maximum variation in patient characteristics, including, in this case, education, EDSS, and area of Italy. A minimum of 10 MS patients was interviewed, and recruitment stopped when data saturation was achieved [22]. Interview content was analysed informally. After providing informed consent, patients completed both the MSQOL-54 and the new short instrument. Within a week of questionnaire completion, a psychologist (AT), experienced at interviewing, conducted individual telephone interviews with patients–who had their completed questionnaires to hand–using a semi-structured interview guide. The aim was to assess the acceptability of the short version in comparison to the original and discover whether any of the removed items was considered important (domain coverage), or any revised response category was considered confusing or unclear.

### Statistical analyses

Continuous data were summarized using means, standard deviations, medians and ranges/interquartile ranges. Categorical data were summarized using frequencies. Correlations were estimated using Pearson’s r (p value of less than 0.05 was considered to be statistically significant). CFA was used to assess the dimensionality and PCM to assess metric properties of the MSQOL-54. First we investigated MSQOL-54 dimensionality by confirmatory factor analysis (CFA) which is recommended over exploratory factor analysis (EFA) when there is an a priori hypothesis regarding dimensionality, as it allows testing of whether the empirical data fit an assumed structure [23].

We considered the models acceptable if the following criteria were met: root mean square error of approximation (RMSEA) <0.08; comparative fit index (CFI) >0.90; and standardized root mean square residual (SRMR) <0.08 [24,25].

We next assessed the fit of the data for each MSQOL-54 subscale to PCM using a joint maximum likelihood estimator.

The models assume unidimensionality–that all items (here HRQOL subscale) assess the same single construct of interest–and also local independence–that items do not correlate with each other when the latent trait has been controlled for.

We used post-hoc principal component analysis (PCA) of residuals to check unidimensionality (i.e. if the one dimension hypothesis was respected, residuals do not contain any significant dimension) and correlation between item residuals to check local independence. We considered the assumptions satisfied if the first eigenvalue of the PCA was ≤2 and all the correlations between item residuals were ≤0.40 [26].

For each subscale reliability was evaluated by the following reliability index: $RI=\frac{{PSI}^{2}}{1+{PSI}^{2}}$, where PSI (person separation index) is the ratio between measure standard deviation and the root mean-square standard error (RMSE). RI values ≥0.70 are considered satisfactory [27].

Next, for each item, the thresholds between response categories were assessed, where a threshold is the point between two response categories at which each response has equally probability of being chosen. If the estimates of response thresholds are properly ordered, a higher response always indicates an increase in the underlying trait (in our case higher functioning). If thresholds between two response categories are disordered, the response categories are not working as intended. This can be remedied in the analyses by merging (in Rasch terminology, ‘collapsing’) the two response categories. In the short questionnaire, for a pair of categories with a disordered threshold, we retained the category that best expressed the order of the Likert scale and eliminated the other. For example, MSQOL-54 item 29 (*Did you feel worn out*?) has six possible Likert-like responses: *All of the time/Most of the time/A good bit of the time/Some of the time/A little of the time/None of the time*. Ordering analysis revealed disordered thresholds for *A good bit of the time/Some of the time*. We removed *A good bit of the time* and were left with: *All of the time/Most of the time/Some of the time/A little of the time/None of the time*.

We next assessed the adequacy of fit of each item by information-weighted (infit) and outlier-sensitive (outfit) statistics, which measure information about responses given by persons with an ‘ability’ level close to (infit) or distant from (outfit) the item difficulty level: values for both of these in the 0.7–1.3 range are considered satisfactory [28–32].

We assessed the differential item functioning (DIF) to determine whether there were differences due to a context effect on the measures [33]. We evaluated DIF for gender, age (two categories, median [40 years] as cut-off), and EDSS score (two categories, median [2.0] as cut-off). A DIF value of least 0.5 logits associated with a p value <0.05 was considered to indicate a significant difference in item difficulty between the categories.

Any misfitting item in term of infit/outfit and DIF was discarded and the analysis re-run. This iterative process was continued until no further misfit was observed.

Item discrimination was also evaluated using point-measure correlation that provides a measure of the correlation between single item scores and PCM measures.

Based on the results of the above analyses we derived the short questionnaire: we assessed its dimensionality by EFA using Kaiser eigenvalue criteria, scree-plot and percentage of explained variance. Dimensionality was also checked by CFA. To maximize comparability with the original instrument, we used the same EFA extraction (principal axis factoring) and rotation (PROMAX) methods [3] as used on the MSQOL-54. Subscales having a factor loading ≥0.35 were selected to contribute to each factor’s composite score. The adequacy of the CFA solution was evaluated by Satorra-Bentler scaled chi-square test [34].

The analyses were performed with SAS release 9.3 (descriptive analyses and EFA), WINSTEPS 3.72.3 Beaverton, OR, USA (PCM), and Lisrel 8.72 (CFA).

## Results

The entire dataset consisted of MSQOL-54s complied by 635 MS patients (mean age 40.8 years, 67% women, median EDSS 2.5, range 0–9.5) whose characteristics are summarized in Table 2. Higher EDSS scores for Rome and Chieti patients are because: (a) Rome patients came from the S. Lucia Foundation, a rehabilitation research hospital that follows severely compromised patients; while (b) Chieti provided data from a cross-sectional study on patients followed at their MS Day Hospital. The other centres provided data on patients with shorter disease duration and less severe compromise. About 25% of compilations had at least one missing response. For each subscale, around 5% of items lacked responses; however, for the subscales Sexual Function and Satisfaction with Sexual Function, 9% to 15% lacked responses (S1 Table). Compilations from 157 patients were excluded from the analyses: 152 because of one or more missing responses on MSQOL-54 and 5 because of missing or invalid EDSS score. These 157 excluded patients were similar in age, EDSS score, and disease duration to included ones, except for a higher proportion of women (75% vs. 65%; p = 0.02). Notably, this difference was specific for the 5 items on sexual function and satisfaction (for the remaining 49 items, women were 66% in excluded vs. 65% in included cases; p = 0.86).

**Table 2:** Characteristics of entire dataset (635 patients) and of the analysis dataset (473 patients) by MS centre.

	Milan^1^ (n = 276)	Orbassano (n = 152)	Catania (n = 97)	Rome (n = 64)	Chieti (n = 46)	Total (n = 635)	Analysed (n = 473)
Women (%)	180 (65.2)	110 (72.4)	65 (67.0)	38 (59.4)	33 (71.7)	426 (67.1)	305 (64.0)
Mean age in years, SD (range)	39.1, 9.6 (19–65)	39.6, 10.3 (18–66)	34.2, 9.4 (18–62)	52.7, 12.3 (24–79)	46.7, 9.8 (29–74)	40.4, 11.2 (18–79)	40.8, 11.3 (18–79)
Mean years from 1st symptoms to MS diagnosis, SD (range)	7 (0–40)	9 (1–32)	5 (1–28)	21 (1–44)	15 (4–38)	9 (0–44)	9 (0–44)
Median EDSS score (range)	2.0 (0–7.0)	2.0 (0–8.5)	2.0 (0–5.5)	7.3 (1.5–9.5)	4.5 (1.0–8.5)	2.5 (0–9.5)	2.0 (0–9.5)
Mean MSQOL-54 PHC, SD (range)	66.4, 19.1 (17–100)	64.5, 20.2 (7–97)	62.2, 19.8 (20–97)	36.3,13.2 (2–77)	58.8, 18.4 (17–92)	61.3, 20.9 (2–100)	61.6, 21.3 (2–100)
Mean MSQOL-54 MHC, SD (range)	63.1, 21.7 (3–99)	65.0, 21.8 (6–99)	58.5, 24.1 (8–97)	47.9, 18.0 (2–81)	64.1, 21.7 (11–97)	61.3, 22.3 (2–99)	61.2, 22.4 (2–99)
Excluded cases^2^ (%)	76 (27.5)	33 (21.7)	17 (17.5)	1 (1.6)	30 (65.2)	157 (24.7)	0 (0.0)

EDSS, Expanded Disability Status Scale; MSQOL-54, Multiple Sclerosis Quality of Life-54; PHC/MHC, Physical and Mental Health Composite; SD standard deviation.

^1^ 150 patients participated in the MAIN trial [35], and 82 in the CRIMS trial [36] (baseline assessment).

^2^ 152 patients with ≥1 missing responses on MSQOL-54, 5 patients with missing or invalid EDSS score

Psychometric analyses were performed on completed MSQOL-54s from 473 patients (S1 Dataset).

### Factor and Rasch analyses on MSQOL-54

CFA of the 12 MSQOL-54 subscales showed good fit overall (RMSEA 0.054; CFI 0.98; SRMR 0.052) (S1 Fig).

Data for each of the 11 MSQOL-54 subscales (50 items) with ≥3 items were fitted to the PCM. For all subscales, post-hoc PCA of residuals yielded a first eigenvalue ≤2, thus in all cases the unidimensional assumption was satisfied. Furthermore in no case the correlation between item residuals was >0.40, satisfying the local independence assumption. RI was ≥0.70 in 7/11 subscales.

Sixteen of the 50 items had disordered thresholds (Table 3); they were therefore ‘collapsed’ and then calibrated. Table A of S1 Appendix reports the PCM results of the original sub-scales before removing any items. After this first analysis, in each sub-scale the worst fitting item was deleted and the PCM re-run. Afterwards, according to the new fit statistics, the next worst item was deleted, and so until no further misfitting item was present. Table B of S1 Appendix reports all deleted items and their diagnostic statistics.

**Table 3:** MSQOL-54 items with disordered thresholds.

Subscale	Number of response categories	Pairs of categories with disordered thresholds	
		Categories 3 and 4	Categories 4 and 5
Health Perceptions	5	Items 35*, 37*	Item 36
Emotional Wellbeing	6	Item 24	Items 26*, 30*
Energy	6	Items 29, 31	Items 23*, 27*, 32*
Cognitive Function	6	Items 42, 44, 45	–
Health Distress	6	Items 40, 41	–

MSQOL-54, Multiple Sclerosis Quality of Life-54.

* Categories refer to reversed items.

In the pruning of Emotional Wellbeing subscale, following the statistical criteria we should have deleted 3 of the 5 items. As this subscale has great clinical relevance (also in terms of MSQOL-54 mental composite score), we removed the two items (24, 28) with borderline infit/outfit statistics.

The subscales Role limitations—Physical (four items) and Role Limitations—Emotional (three items) had an RI of 0. The RI of the remaining three subscales was unsatisfactory: 0.69 (borderline) for Sexual Function, 0.63 for Social Function (three items), and 0.62 for Health Perceptions (three items).

In fact the role limitation subscales suffered from marked floor or ceiling effects with minimum or maximum score for Role Limitations—Physical obtained by 70%, and minimum or maximum score for Role Limitations—Emotional obtained by 78%. Both these scales have few items and each has only two response categories. These characteristics were responsible for the zero reliability and led us to exclude them from the short instrument.

The expert panel considered that although the Sexual Function, Social Function and Health Perceptions subscales had an unsatisfactory RI, they investigated important HRQOL dimensions for people with MS and were therefore retained, two in modified form [1,2,12,37]. Thus, all four items were retained in Sexual Function, while for Social Function and Health Perceptions, a single item was retained (chosen based on item’s statistical fit, content and wording): item 33 (*During the past 4 weeks*, *how much of the time has your physical health or emotional problems interfered with your social activities [like visiting with friends*, *relatives*, *etc*.*]*?*)* for Social Function, and item 35 (*I am as healthy as anybody I know*) for Health Perceptions.

We consider now the MSQOL-54 subscales with ≤2 items, which could not be analysed by PCM. The Overall Quality of Life subscale has two items, both of which address the same question. We retained item 53 (visual analogue scale) and eliminated item 54. Of the two single-item subscales, Change in Health was retained because of its clinical importance for retest assessment, while Satisfaction with Sexual Function (item 50) was removed because it overlaps with Sexual Function (items 46–49) (Table 4): in particular with item 49 (*Ability to satisfy sexual partner*) which pertains to both functioning and satisfaction. All four Sexual Function items (46–49) were preserved.

**Table 4:** Structural comparison of MSQOL-54 and MSQOL-29, with MSQOL-29 psychometric characteristics.

MSQOL-54			MSQOL-29				
Subscale	No. of items	Item number	Subscale	No. of items	Item number	Reliability index ^^^	Dimensionality score^§^
Physical Function	10	3–12	Physical Function	6	4, 5, 6, 7, 9, 11	0.81	1.7
Role Limitations—Physical	4	13–16	–	–	–	–	–
Role Limitations—Emotional	3	17–19	–	–	–	–	–
Bodily Pain	3	21, 22, 52	Bodily Pain	3	Same as original	0.85	1.6
Emotional Wellbeing	5	24–26, 28, 30	Emotional Wellbeing	3	25, 26^C^, 30^C^	0.79	1.6
Energy	5	23, 27, 29, 31, 32	Energy	3	27^C^,29^C^, 31^C^	0.81	1.7
Health Perceptions	5	1, 34–37	Health Perceptions	1	35^C^		
Social Function	3	20, 33, 51	Social Function	1	33		
Cognitive Function	4	42–45	Cognitive Function	3	42^C^, 43, 44^C^	0.78	1.7
Health Distress	4	38–41	Health Distress	3	38, 39, 41^C^	0.86	1.6
Sexual Function	4	46–49	Sexual Function	4	Same as original	0.69	1.4
Change in Health	1	2	Change in Health	1	Same as original		
Satisfaction with Sexual Function	1	50	–	–	–	–	–
Overall Quality of Life	2	53, 54	Overall Quality of Life	1	53		

MSQOL-54, Multiple Sclerosis Quality of Life-54; MSQOL-29, Multiple Sclerosis Quality of Life-29.

^C^ Items with collapsed response categories

^^^ Values ≥0.70 indicate acceptable reliability

^§^ Values ≤2.0 indicate unidimensionality

In summary, 25 items were removed: 7 had reliability index of zero, 5 had infit/outfit misfit, 5 had DIF misfit, 3 had infit/outfit and DIF misfit, 2 had low reliability, and 3 for content considerations. The resulting short version (MSQOL-29) was made of 29 items.

### The short instrument

The 29 items of MSQOL-29 (54% of MSQOL-54) were grouped into 7 multi-item and 4 single-item subscales (Table 4). A filter question (*During the past 4 weeks*, *have you had an active sexual life*?) was added after the first Sexual Function item. If the reply is “no” these items are skipped, and in the electronic version the questions are not shown. Table 5 shows PCM measurement estimates and item fit statistics (infit and outfit) for the MSQOL-29 multi-item subscales. The PCM logit measure column reports each item’s difficulty (higher logits corresponding to more ‘difficult’ items), and the τ1–5 columns are the category thresholds. Item fit statistics for all the MSQOL-29 multi-item subscales were within acceptable ranges. Point measure correlation values were satisfactory, as they ranged between 0.84 and 0.95 (Table 5),

**Table 5:** Partial credit measurement indexes for MSQOL-29 items (and subscales) retained in the final model.

Item no.	Logit measure (SE)	Infit*	Outfit*	Point measure correlation	τ1	τ2	τ3	τ4	τ5
**Physical Function**									
9	1.62 (0.13)	1.11	1.09	0.89	-1.72	1.72	–	–	–
4	0.68 (0.14)	0.95	0.94	0.91	-2.29	2.29	–	–	–
6	0.59 (0.14)	0.88	0.87	0.92	-2.69	2.69	–	–	–
5	0.31 (0.13)	1.09	1.11	0.90	-2.02	2.02	–	–	–
7	-1.34 (0.15)	0.92	0.93	0.90	-1.96	1.96	–	–	–
11	-1.86 (0.15)	1.04	0.83	0.87	-1.47	1.47	–	–	–
**Bodily Pain**									
21	0.93 (0.10)	0.90	0.83	0.95	-7.34	-3.30	1.48	2.80	6.30
22	-0.24 (0.11)	0.86	0.76	0.93	-5.82	-2.60	3.12	5.30	–
52	-0.69 (0.12)	1.05	1.02	0.92	-6.44	-1.90	2.75	5.60	–
**Emotional Wellbeing**									
26^c^	0.63 (0.09)	0.96	0.92	0.85	-5.47	-2.40	1.15	6.70	–
30^c^	0.35 (0.09)	1.04	1.02	0.84	-5.42	-2.20	1.60	6.00	–
25	-0.98 (0.07)	0.93	0.95	0.86	-3.72	-1.70	-0.90	1.70	4.70
**Energy**									
31^c^	1.07 (0.09)	0.76	0.78	0.89	-4.68	-2.80	2.14	5.40	–
27^c^	0.42 (0.09)	1.20	1.21	0.85	-5.41	-1.60	1.37	5.70	–
29^c^	-1.49 (0.09)	0.90	0.88	0.88	-5.12	-2.00	2.14	5.00	–
**Cognitive Function**									
44^c^	0.21 (0.10)	1.21	1.16	0.86	-4.21	-2.50	1.09	5.60	–
43	-0.10 (0.08)	0.90	0.91	0.89	-3.78	-1.70	-1.20	1.40	5.30
42^c^	-0.11 (0.10)	0.82	0.81	0.91	-3.89	-2.50	1.47	5.00	–
**Health Distress**									
38	0.29 (0.09)	0.97	0.99	0.93	-5.54	-2.10	-1.50	2.30	6.80
41^c^	0.03 (0.09)	1.09	1.08	0.91	-3.44	-2.20	1.17	4.50	–
39	-0.32 (0.08)	0.75	0.73	0.94	-4.68	-1.50	-1.10	2.10	5.10
**Sexual Function**									
46	0.04 (0.10)	1.10	1.09	0.89	-1.99	-0.10	2.04	–	–
47	0.01 (0.10)	0.86	0.86	0.90	-1.64	-0.30	1.90	–	–
48	-0.01 (0.10)	0.98	0.96	0.89	-1.60	-0.30	1.86	–	–
49	-0.04 (0.10)	1.02	0.99	0.88	-1.33	-0.20	1.55	–	–

MSQOL-29, Multiple Sclerosis Quality of Life-29. For each subscale, items are reported in measurement order (more difficult at top); 1-5 are category thresholds.

*Values in the range 0.7–1.3 indicate good fit

^c^ Items with collapsed response categories

DIF analysis of MSQOL-29 indicated a significant difference in functioning across age for item 26 (*Have you felt calm and peaceful*?), the remaining items did not have DIF for age, gender or EDSS score (S2 Table).

An English version of the MSQOL-29 is provided in the S2 Appendix.

### MSQOL-29 Mental and Physical Health Composites

EFA of MSQOL-29 resulted in two factors according to the eigenvalue criteria (5.323 and 1.045), even if the scree plot suggested to retain one factor. The one- and two-factor solutions accounted for 44.2% and 49.8% of the total variance respectively. The CFA of the two-factor solution indicated reasonably adequate fit (RMSEA 0.065; CFI 0.98; SRMR 0.04) and outperformed the one-factor solution (Satorra-Bentler scaled chi-square test [3] = 137.5; p <0.001). The two-factor solution was also adopted in order to preserve the original dimensionality of the questionnaire, together with domain interpretability. Two composite factors (MHC and PHC) were derived by analogy with those of the MSQOL-54 (Table 6).

**Table 6:** PROMAX rotated two-factor solution for MSQOL-29 subscales and weightings for the Mental (MHC) and Physical (PHC) Health Composite scores.

Subscale	Standardized regression coefficient (loading)		Weightings	
	MHC	PHC	MHC	PHC
Bodily Pain	0.41	0.07	0.11	0
Emotional Wellbeing	0.93	-0.09	0.26	0
Cognitive Function	0.68	-0.07	0.19	0
Social Function (single item)	0.67	0.14	0.19	0
Energy	0.52	0.38	0.14	0.10
Health Distress	0.39	0.51	0.11	0.14
Physical Function	-0.18	1.01	0	0.27
Sexual Function	0.15	0.29	0	0.08
Health Perceptions (single item)	-0.01	0.48	0	0.13
Overall Quality of Life (single item)	0.15	0.72	0	0.19
Change in Health (single item)	0.12	0.38	0	0.10

MSQOL-29, Multiple Sclerosis Quality of Life-29.

The factor MHC comprised, in order of saturation: Emotional Wellbeing, Cognitive Function, Social Function (item 33: *During the past 4 weeks*, *how much of the time has your physical health or emotional problems interfered with your social activities [like visiting with friends*, *relatives*, *etc*.*]*?*)*, Energy, Bodily Pain, and Health Distress, with loadings in the 0.93–0.39 range. The factor PHC comprised: Physical Function, Overall Quality of Life, Health Distress, Health Perceptions (item 35: *I am as healthy as anybody I know*), Energy, Change in Health (item 2: *Compared to one year ago*, *how would you rate your health in general now*?), and Sexual Function, with loadings in the 1.01–0.29 range.

The MSQOL-29 MHC and PHC had the same marker variables (Emotional Wellbeing and Physical Function respectively) as obtained for the original MSQOL-54 analysis [3] with a correlation between them of 0.72 (0.66 originally). Health Distress and Energy had above-threshold loads on both factors (which was the case only for Health Distress in the original MSQOL-54 analysis). PHC and MHC weightings were obtained based on subscale loadings: for loadings <0.29 a weighting of 0 was assigned, and the remaining values were re-scaled so that their sum was 1.0 (Table 6).

### Comparison of short and original instruments

Table 7 shows MSQOL-29 subscale and composite scores compared to those of MSQOL-54. Mean values of MSQOL-29 subscale and composite scores were close to those of the original questionnaire, the greatest difference being for Cognitive Function (mean 70.2 for MSQOL-54; 66.7 for MSQOL-29). Differences between the composites were as follows: Bodily Pain and Social Function belong to the MHC in MSQOL-29 and not to the PHC; Overall Quality of Life belongs to the PHC in MSQOL-29 and not to the MHC; Energy belongs to both composites (and not to PHC only as in MSQOL-54) (Table 6).

**Table 7:** MSQOL-54 and MSQOL-29: mean subscale scores with standard deviations (SD) and Pearson’s correlations.

	MSQOL-54	MSQOL-29	
Subscale	Mean score (SD) median (interquartile range)		Correlation (Pearson’s r ^§^)
Physical Function	63.3 (35.1) 75.0 (40–95)	63.7 (36.4) 75.0 (33–100)	0.99
Bodily Pain*	76.7 (25.2) 85.0 (55–100)	–	–
Emotional Wellbeing	59.1 (21.6) 60.0 (44–76)	60.4 (21.0) 60.0 (45–76)	0.96
Energy	51.1 (22.2) 48.0 (36–68)	51.7 (23.1) 50.0 (33–67)	0.94
Cognitive Function	70.2 (24.1) 75.0 (55–90)	66.7 (24.3) 68.3 (53–85)	0.97
Health Distress	60.8 (29.8) 65.0 (40–85)	62.2 (30.4) 70.0 (43–87)	0.99
Sexual Function*	75.3 (31.6) 91.7 (58–100)	–	–
Physical Health Composite (PHC)	61.6 (21.3) 63.0 (45–80)	58.8 (22.7) 62.8 (44–78)	0.92
Mental Health Composite (MHC)	61.2 (22.4) 62.7 (44–80)	62.1 (19.6) 63.0 (49–80)	0.91

MSQOL-29, Multiple Sclerosis Quality of Life-29.

* MSQOL-29 items identical to MSQOL-54 items.

^§^ All p values for Pearson’s r <0.001.

### Cognitive debriefing

Twelve patients were interviewed (5 men, age range 21–68 years, EDSS range 0–7.5, 3 living in northern, 4 in central, and 5 in southern Italy). All considered that the MSQOL-29 was easy to complete, and 11/12 preferred it because it was less demanding without losing any important content. The reduced number of categories for 9 items was not thought to adversely affect clarity or ease of selecting a response. One person (47 year-old woman) preferred the MSQOL-54: she thought it covered more domains, and the greater number of alternative replies made it easier for her to find a response exactly matching her opinion. Twenty-four of the 25 removed items were considered expendable; two interviewees (a 37 year-old man and a 32 year-old woman) considered that the single item subscale Sexual Satisfaction was important for the overall evaluation and should not have been removed.

## Discussion

We shortened the MSQOL-54 using a combination of psychometric analyses (factor analysis and Rasch modelling) and input from MS/HRQOL professionals and MS patients. The resulting MSQOL-29 consists of 7 multi-item and 4 single-item subscales, used to form two composites (PHC, MHC), consistent with the theoretical construct used to develop the original instrument [38].

The new instrument requires approximately 10 minutes to complete, considerably less than the 19 minutes required for MSQOL-54 [5]. Nonetheless, the high correlation of MSQOL-29 subscale and composite scores with those of MSQOL-54 (Table 7) suggests that eliminating items and subscales did not substantially change the HRQOL dimensions found for the original instrument. Confirmation of these findings in an independent sample (second phase of the project) is however needed.

The most conspicuous change is that the Physical and Emotional Role Limitation subscales (Table 1), are not present in MSQOL-29. These were eliminated because both had maximum or minimum scores in 70% or more cases. Similar findings for these subscales have been reported elsewhere [39,40]. Role Limitations—Physical and Emotional were in fact considered expendable by all patient interviewees.

We also removed the single-item subscale Satisfaction with Sexual Function, because sex was adequately investigated by the four items of Sexual Function (although two interviewees did not agree).

To the best of our knowledge, PCM has not been previously applied to the MSQOL-54. Rasch modelling was originally designed and used for educational assessment, but is increasingly used in health research as it has advantages over factor analysis. Unlike the most widely used statistical approaches which describe data by fitting models to them, Rasch analysis evaluates the fundamental scaling properties of an instrument to determine whether it has the properties prescribed by the axiomatic Rasch model [41]. Rasch analysis has gained wide acceptance in the medical literature as a gold standard for refining existing scales, constructing new scales conforming to the Rasch model, and measuring people’s traits in educational, social and biomedical sciences. We used a model of the Rasch family principally for its ability to reduce the number of items while retaining the instrument’s psychometric properties [42]. Note however that the two composite scales (PHC and MHC) of the MSQOL-29 conform to the original additive model, and cannot be treated as ‘Rasch measures’.

As regards the size of the database of completed MSQOL-54s, this exceeded the minimum of 250 compilations recommended by de Ayala [43]. We decided to analyse only MSQOL-54s with all items completed, and for which age, gender, diagnosis, and EDSS were available, even though Rasch modelling can cope with missing items. Adopting this policy meant that our database remained constant for all analyses.

Of the 157 (25%) MSQOL-54 compilations with missing responses, 113 (72%) concerned sexuality (Sexual Function and Satisfaction with Sexual Function), and women had higher proportion of missing replies to such items (n = 90/426, 21%) compared to men (n = 23/209, 11%; p = 0.002). High levels of missing responses for the MSQOL-54 sex subscales have been repeatedly reported [5,8]. Reasons may be embarrassment, because the issue did not arise within the one-month referral period, and lack of clarity. Regarding the latter point, Catherine Acquadro reported that item 47 –the only MSQOL-54 item with a different wording in men (*difficulty getting or keeping an erection*) and in women (*inadequate lubrication*) was difficult to understand for women (personal communication, 2015). We found the highest proportion of missing replies in women (15%, S1 Table) on this item.

Missing items can introduce bias and lack of power in data analysis, and existing methods to impute missing data are far from perfect [44]. Nevertheless, we retained the Sexual Function items as they address a key HRQOL domain [1–3,12,37]. We added a filter question after the first Sexual Function item so that subsequent subscale items can be skipped if not pertinent–to thereby reduce the burden of compilation (S2 Appendix) and distinguish between missed and non-pertinent items.

A limitation of the study is that we did not extensively revise the instrument in passing to the short version: our aim was to work with the original set of items and subscales, and retain those that had the best psychometric characteristics. Specifically, we did not revise any item wording, only ‘collapsing’ response categories (for 9 items) when thresholds were disordered. Although some malfunctioning subscales were identified (Social Function and Health Perceptions), they were retained as single-item subscales because of the importance of these domains as indicated by feedback from clinicians and patients.

We analysed MSQOL-54s from a heterogeneous sample of people with MS, so that the MSQOL-29 is likely to be applicable to a wide range of MS patients, all of whom were Italian. Nevertheless since MSQOL-29 is only a shortened form of MSQOL-54 it is also likely to be easily applicable to MS patients from different countries and cultures. However, international multicultural initiatives are likely to be the way forward for developing and revising patient-reported instruments in the future.

The MSQOL-29 is now being evaluated on a large (500) independent sample of people with MS from the three geographic areas of Italy with the aim of investigating its construct and discriminant validity, reliability and responsiveness [14,45]. The equivalence of the paper and electronic versions will be also assessed [46].

## Supporting Information

S1 Appendix Partial Credit Model results on MSQOL-54 and on its deleted items. (DOCX)

S2 Appendix English version of the MSQOL-29 questionnaire. (DOCX)

S1 Dataset Raw data from patients’ case record form, and MSQOL-54 included in the present analysis (473 patients). (XLS)

S1 Fig Confirmatory factor analysis of the MSQOL-54. (PDF)

S1 Table Distribution of missing responses on MSQOL-54 Sexual Function and Satisfaction with Sexual Function subscales by gender. (PDF)

S2 Table Differential item functioning of the MSQOL-29. (PDF)
