# Supplementary material for: Development of a Short Version of MSQOL-54 Using Factor Analysis and Item Response Theory
Source: PLoS One. 2016 Apr 14;11(4):e0153466. doi: 10.1371/journal.pone.0153466 (PMC4831784; doi:10.1371/journal.pone.0153466)
Supplement: S1 Appendix — (DOCX) [file pone.0153466.s001.docx]

**S1 Appendix**

**Table A.** Partial credit model statistics on MSQOL-54 subscales with more than two items.

| **Item** | **Logit measure (SE)** | **Infit** | **Outfit** | | **Gender** | | | | | **Age** | | | **EDSS score** | | |
| --- | --- | --- | --- | --- | --- | --- | --- | --- | --- | --- | --- | --- | --- | --- | --- |
|  |  |  |  |  | **DIF** | | **p** | | | **DIF** | | **p** | **DIF** | | **p** |
| **Physical Function** | | | | | | | | | | | | | | | |
| RI=0.86 First PCA eigenvalue=1.8 | | | | | | | | | | | | | | | |
| 3 | 3.69 (0.14) | 1.37 | 1.56 | | 0.41 | | 0.21 | | | -0.20 | | 0.21 | -0.08 | | 0.18 |
| 4 | 0.55 (0.13) | 0.89 | 0.75 | | -0.07 | | 0.78 | | | -0.42 | | 0.24 | -0.38 | | 0.33 |
| 5 | 0.23 (0.13) | 1.07 | 0.98 | | -0.52 | | 0.07 | | | 0.13 | | 0.58 | 0.40 | | 0.13 |
| 6 | 0.45 (0.13) | 0.87 | 0.74 | | -0.25 | | 0.31 | | | 0.12 | | 0.52 | -0.03 | | 0.75 |
| 7 | -1.23 (0.14) | 0.93 | 0.72 | | -0.03 | | 0.75 | | | -0.11 | | 0.72 | 0.33 | | 0.26 |
| 8 | 0.10 (0.13) | 1.06 | 1.11 | | 0.00 | | 0.87 | | | 0.17 | | 0.55 | 0.64 | | 0.04 |
| 9 | 1.37 (0.12) | 0.97 | 0.92 | | -0.07 | | 0.95 | | | -0.20 | | 0.37 | -0.17 | | 0.53 |
| 10 | -0.74 (0.13) | 0.79 | 0.67 | | 0.40 | | 0.09 | | | 0.33 | | 0.11 | -0.52 | | 0.23 |
| 11 | -1.68 (0.14) | 0.88 | 0.59 | | 0.14 | | 0.80 | | | 0.11 | | 0.46 | -0.26 | | 0.62 |
| 12 | -2.74 (0.16) | 1.18 | 2.52 | | 0.20 | | 0.59 | | | 0.13 | | 0.86 | -0.26 | | 0.43 |
| **Bodily Pain** | | | | | | | | | | | | | | | |
| RI=0.85 First PCA eigenvalue=1.6 | | | | | | | | | | | | | | | |
| 21 | 0.93 (0.10) | 0.90 | 0.83 | | 0.25 | | | 0.20 | | -0.03 | | 0.76 | -0.04 | | 0.86 |
| 22 | -0.24 (0.11) | 0.86 | 0.76 | | 0.03 | | | 0.73 | | 0.15 | | 0.28 | 0.08 | | 0.66 |
| 52 | -0.69 (0.12) | 1.05 | 1.02 | | -0.40 | | | 0.09 | | -0.12 | | 0.51 | -0.04 | | 0.82 |
| **Emotional Wellbeing** | | | | | | | | | | | | | | | |
| RI=0.87 First PCA eigenvalue=1.7 | | | | | | | | | | | | | | | |
| 24^c^ | 0.30 (0.09) | 1.30 | | 1.30 | | -0.47 | | | 0.01 | | 0.38 | 0.09 | | 0.27 | 0.58 |
| 26^c^ | 0.51 (0.09) | 0.99 | | 1.07 | | -0.19 | | | 0.31 | | 0.57 | 0.00 | | 0.00 | 0.88 |
| 30^c^ | 0.27 (0.09) | 1.16 | | 1.14 | | 0.02 | | | 0.73 | | 0.07 | 0.85 | | 0.39 | 0.10 |
| 28 | -0.24 (0.07) | 0.71 | | 0.70 | | 0.12 | | | 0.26 | | -0.31 | 0.02 | | -0.23 | 0.35 |
| 25 | -0.83 (0.07) | 0.77 | | 0.76 | | 0.27 | | | 0.03 | | -0.34 | 0.00 | | -0.22 | 0.22 |
| **Energy** | | | | | | | | | | | | | | | |
| RI=0.82 First PCA eigenvalue=1.8 | | | | | | | | | | | | | | | |
| 31^c^ | 0.81 (0.07) | 0.79 | | 0.80 | | 0.00 | | | 0.64 | | -0.22 | 0.32 | | -0.47 | 0.00 |
| 27^c^ | 0.38 (0.07) | 0.77 | | 0.76 | | 0.33 | | | 0.00 | | -0.31 | 0.04 | | -0.26 | 0.32 |
| 23^c^ | -0.11 (0.08) | 1.01 | | 1.00 | | 0.30 | | | 0.14 | | -0.11 | 0.36 | | 0.14 | 0.21 |
| 32^c^ | -0.27 (0.07) | 1.53 | | 1.52 | | -0.39 | | | 0.01 | | 0.83 | 0.00 | | 0.89 | 0.00 |
| 29^c^ | -0.81 (0.07) | 0.86 | | 0.83 | | -0.21 | | | 0.26 | | -0.24 | 0.13 | | -0.32 | 0.01 |
| **Cognitive Function** | | | | | | | | | | | | | | | |
| RI=0.79 First PCA eigenvalue=1.9 | | | | | | | | | | | | | | | |
| 44^c^ | 0.35 (0.08) | 0.84 | | 0.83 | | -0.16 | | | 0.60 | | -0.17 | 0.13 | | -0.08 | 0.74 |
| 43 | 0.11 (0.07) | 0.99 | | 1.02 | | 0.15 | | | 0.27 | | 0.00 | 0.89 | | -0.08 | 0.59 |
| 42^c^ | 0.10 (0.08) | 0.86 | | 0.87 | | -0.17 | | | 0.36 | | 0.23 | 0.09 | | -0.08 | 0.90 |
| 45^c^ | -0.55 (0.08) | 1.29 | | 1.43 | | 0.09 | | | 0.10 | | -0.08 | 0.73 | | 0.23 | 0.37 |
| **Health Distress** | | | | | | | | | | | | | | | |
| RI=0.88 First PCA eigenvalue=1.5 | | | | | | | | | | | | | | | |
| 38 | -0.15 (0.08) | 0.88 | | 0.87 | | -0.12 | | | 0.40 | | -0.07 | 0.53 | | -0.23 | 0.23 |
| 41^c^ | -0.29 (0.09) | 1.03 | | 1.05 | | -0.19 | | | 0.29 | | -0.11 | 0.54 | | 0.18 | 0.25 |
| 39 | -0.60 (0.08) | 0.80 | | 0.76 | | 0.11 | | | 0.52 | | -0.24 | 0.17 | | -0.25 | 0.18 |
| 40^c^ | 1.04 (0.10) | 1.10 | | 1.09 | | 0.24 | | | 0.16 | | 0.57 | 0.01 | | 0.41 | 0.15 |
| **Sexual Function** | | | | | | | | | | | | | | | |
| RI=0.69 First PCA eigenvalue=1.4 | | | | | | | | | | | | | | | |
| 46 | 0.04 (0.10) | 1.10 | | 1.09 | | -0.40 | | | 0.15 | | 0.30 | 0.16 | | 0.04 | 0.88 |
| 47 | 0.01 (0.10) | 0.86 | | 0.86 | | 0.36 | | | 0.09 | | -0.11 | 0.65 | | 0.13 | 0.47 |
| 48 | -0.01 (0.10) | 0.98 | | 0.96 | | -0.37 | | | 0.04 | | -0.06 | 0.74 | | -0.15 | 0.54 |
| 49 | -0.04 (0.10) | 1.02 | | 0.99 | | 0.37 | | | 0.05 | | -0.12 | 0.51 | | -0.03 | 0.93 |
| **Role Limitations - Physical** | | | | | | | | | | | | | | | |
| RI=0.00 First PCA eigenvalue=1.4 | | | | | | | | | | | | | | | |
| 13 | -1.48 (0.22) | 1.08 | | 1.22 | | 0.95 | | | 0.05 | | -0.05 | 0.99 | | 0.09 | 0.94 |
| 14 | 0.73 (0.19) | 1.07 | | 1.07 | | -0.46 | | | 0.25 | | 0.65 | 0.10 | | 0.04 | 0.95 |
| 15 | 0.18 (0.19) | 0.77 | | 0.74 | | 0.10 | | | 0.62 | | 0.00 | 0.65 | | -0.24 | 0.68 |
| 16 | 0.58 (0.19) | 1.08 | | 1.11 | | -0.38 | | | 0.33 | | -0.55 | 0.20 | | 0.12 | 0.84 |
| **Role Limitations - Emotional** | | | | | | | | | | | | | | | |
| RI=0.00 First PCA eigenvalue=1.6 | | | | | | | | | | | | | | | |
| 17 | -1.20 (0.24) | 1.09 | | 1.18 | | 0.16 | | | 0.72 | | 0.88 | 0.09 | | 0.12 | 0.93 |
| 18 | -0.08 (0.22) | 0.78 | | 0.78 | | -0.58 | | | 0.11 | | -0.30 | 0.43 | | -0.80 | 0.08 |
| 19 | 1.28 (0.24) | 1.10 | | 1.22 | | 0.56 | | | 0.27 | | -0.47 | 0.35 | | 0.85 | 0.14 |
| **Health Perceptions** | | | | | | | | | | | | | | | |
| RI=0.73 First PCA eigenvalue=1.7 | | | | | | | | | | | | | | | |
| 1 | 0.91 (0.07) | 0.85 | | 0.85 | | 0.00 | | | 0.78 | | -0.64 | 0.00 | | -0.73 | 0.00 |
| 34 | -1.02(0.06) | 1.23 | | 1.11 | | -0.09 | | | 0.57 | | 0.27 | 0.10 | | 0.20 | 0.48 |
| 35^c^ | 0.19 (0.07) | 0.97 | | 0.95 | | 0.09 | | | 0.53 | | 0.44 | 0.00 | | 0.08 | 0.40 |
| 36^c^ | -0.98(0.07) | 1.21 | | 1.35 | | 0.23 | | | 0.21 | | -0.22 | 0.10 | | 0.33 | 0.19 |
| 37^c^ | 0.89 (0.07) | 0.79 | | 0.75 | | -0.16 | | | 0.27 | | -0.07 | 0.90 | | 0.00 | 0.31 |
| **Social Function** | | | | | | | | | | | | | | | |
| RI=0.63 First PCA eigenvalue=1.8 | | | | | | | | | | | | | | | |
| 20 | 0.18 (0.07) | 0.82 | | 0.81 | | -0.03 | | | 0.87 | | 0.47 | 0.00 | | 0.21 | 0.05 |
| 33 | 0.72 (0.08) | 0.93 | | 0.91 | | -0.24 | | | 0.09 | | 0.22 | 0.10 | | 0.39 | 0.00 |
| 51 | -0.90 (0.07) | 1.26 | | 1.52 | | 0.25 | | | 0.22 | | -0.71 | 0.00 | | -0.75 | 0.00 |

DIF, Differential Item Functioning; EDSS, Expanded Disability Status Scale; MSQOL-54, Multiple Sclerosis Quality Of Life-54; PCA, Principal Component Analysis; RI, Reliability Index; ^C^ Items with collapsed response categories

**Table B.** Information about the 25 items deleted.

| **Item deleted** | **Logit measure (SE)** | **Infit** | **Outfit** | **Gender** | | | **Age** | | **EDSS score** | |
| --- | --- | --- | --- | --- | --- | --- | --- | --- | --- | --- |
|  |  |  |  | **DIF** | **p** | | **DIF** | **p** | **DIF** | **P** |
| **Physical Function** | | | | | | | | | | |
| 8 | 0.10 (0.13) | 1.06 | 1.11 | 0.00 | 0.87 | | 0.17 | 0.55 | **0.64** | **0.04** |
| 3 | 3.82 (0.14) | **1.37** | **1.54** | 0.45 | 0.22 | | -0.14 | 0.35 | 0.03 | 0.25 |
| 12 | -2.41 (0.17) | 1.21 | **1.57** | 0.25 | 0.71 | | 0.12 | 0.91 | -0.15 | 0.41 |
| 10 | -0.69 (0.14) | 0.82 | 0.71 | **0.53** | **0.03** | | 0.38 | 0.08 | -0.49 | 0.51 |
| **Emotional Wellbeing** | | | | | | | | | | |
| 24^c^ | 0.30 (0.09) | **1.30** | **1.30** | -0.47 | | 0.01 | 0.38 | 0.09 | 0.27 | 0.58 |
| 28 | -0.24 (0.07) | **0.71** | **0.70** | 0.12 | | 0.26 | -0.31 | 0.02 | -0.23 | 0.35 |
| **Energy** | | | | | | | | | | |
| 32^c^ | -0.27 (0.07) | **1.53** | **1.52** | -0.39 | | 0.01 | **0.83** | **0.00** | **0.89** | **0.00** |
| 23^c^ | -0.27 (0.09) | 1.22 | 1.19 | 0.27 | | 0.23 | 0.15 | 0.60 | **0.52** | **0.02** |
| **Cognitive Function** | | | | | | | | | | |
| 45^c^ | -0.55 (0.08) | 1.29 | **1.43** | 0.09 | | 0.10 | -0.08 | 0.73 | 0.23 | 0.37 |
| **Health Distress** | | | | | | | | | | |
| 40^c^ | 1.04 (0.10) | 1.10 | 1.09 | 0.24 | | 0.16 | **0.57** | **0.01** | 0.41 | 0.15 |
|  | | | | | | | | | | |
| **Role Limitations - Physical RI=0.00** | | | | | | | | | | |
| **Role Limitations - Emotional RI=0.00** | | | | | | | | | | |
|  | | | | | | | | | | |
| **Health Perceptions** | | | | | | | | | | |
| 1 | 0.91 (0.07) | 0.85 | 0.85 | 0.00 | | 0.78 | **-0.64** | **0.00** | **-0.73** | **0.00** |
| 36^c^ | -0.80 (0.07) | 1.20 | **1.40** | 0.24 | | 0.20 | **-0.35** | **0.02** | 0.17 | 0.61 |
| 34 | Deleted by content consideration and low reliability (RI=0.62) | | | | | | | | | |
| 37 |  |  |  |  |  |  |  |  |  |  |
| **Social Function** | | | | | | | | | | |
| 51 | -0.90 (0.07) | 1.26 | **1.52** | 0.25 | | 0.22 | **-0.71** | **0.00** | **-0.75** | **0.00** |
| 20 | Deleted by content consideration | | | | | | | | | |
| **Sexual Satisfaction** | | | | | | | | | | |
| 50 | Deleted by content consideration | | | | | | | | | |
| **Overall Quality of Life** | | | | | | | | | | |
| 54 | Deleted by content consideration | | | | | | | | | |

In bold values of criteria used to remove items. DIF, Differential Item Functioning; EDSS, Expanded Disability Status Scale; ^C^ Items with collapsed response categories; RI, Reliability Index.
