# Supplementary material for: Development of a Short Version of MSQOL-54 Using Factor Analysis and Item Response Theory
Source: PLoS One. 2016 Apr 14;11(4):e0153466. doi: 10.1371/journal.pone.0153466 (PMC4831784; doi:10.1371/journal.pone.0153466)

**S2 Appendix**

**MSQOL-29**

**INSTRUCTIONS**: This survey asks about your health and daily activities. Answer every question by circling the appropriate number (1, 2, 3 ...).

**If you are unsure about how to answer a question, please give the best answer you can and write a comment or explanation in the margin.**

Please feel free to ask someone to assist you if you need help reading or marking the form.

1. **Compared to one year ago**, how would you rate your health in general **now**?

(Circle one number)

| Much better now than one year ago . . . . . . . . . . . . . . . . . . . . . | 1 |
| --- | --- |
| Somewhat better now than one year ago. . . . . . . . . . . . . . . . . | 2 |
| About the same . . . . . . . . . . . . . . . . . . . . . . . . . . . . . . . . . . . .. | 3 |
| Somewhat worse now than one year ago. . . . . . . . . . .. . . . . . | 4 |
| Much worse now than one year ago. . . . . . . . . . . . . . . . . . . . . | 5 |

The following questions are about activities you might do during a typical day. Does **your health** limit you in these activities? If so, how much?

(Circle 1, 2, or 3 on each line)

1. Moderate activities, such as moving a table, pushing a vacuum cleaner, bowling, or playing golf

(Circle one number)

| YES, limited a lot . . . . . . . . . . . . . . . . . . . . . . . . . . . . . . . . . . .. | 1 |
| --- | --- |
| YES, limited a little. . . . . . . . . . . . . . . . . . . . . . . . . . . . . . . . . .. | 2 |
| NO, not limited at all . . . . . . . . . . . . . . . . . . . . . . . . . . . . . . . . . | 3 |

1. Lifting or carrying groceries

(Circle one number)

| YES, limited a lot . . . . . . . . . . . . . . . . . . . . . . . . . . . . . . . . . . .. | 1 |
| --- | --- |
| YES, limited a little. . . . . . . . . . . . . . . . . . . . . . . . . . . . . . . . . .. | 2 |
| NO, not limited at all . . . . . . . . . . . . . . . . . . . . . . . . . . . . . . . . . | 3 |

1. Climbing several flights of stairs

(Circle one number)

| YES, limited a lot . . . . . . . . . . . . . . . . . . . . . . . . . . . . . . . . . . .. | 1 |
| --- | --- |
| YES, limited a little. . . . . . . . . . . . . . . . . . . . . . . . . . . . . . . . . .. | 2 |
| NO, not limited at all . . . . . . . . . . . . . . . . . . . . . . . . . . . . . . . . . | 3 |

1. Climbing one flight of stairs

(Circle one number)

| YES, limited a lot . . . . . . . . . . . . . . . . . . . . . . . . . . . . . . . . . . .. | 1 |
| --- | --- |
| YES, limited a little. . . . . . . . . . . . . . . . . . . . . . . . . . . . . . . . . .. | 2 |
| NO, not limited at all . . . . . . . . . . . . . . . . . . . . . . . . . . . . . . . . . | 3 |

1. Walking more than a mile

(Circle one number)

| YES, limited a lot . . . . . . . . . . . . . . . . . . . . . . . . . . . . . . . . . . .. | 1 |
| --- | --- |
| YES, limited a little. . . . . . . . . . . . . . . . . . . . . . . . . . . . . . . . . .. | 2 |
| NO, not limited at all . . . . . . . . . . . . . . . . . . . . . . . . . . . . . . . . . | 3 |

1. Walking one block

(Circle one number)

| YES, limited a lot . . . . . . . . . . . . . . . . . . . . . . . . . . . . . . . . . . .. | 1 |
| --- | --- |
| YES, limited a little. . . . . . . . . . . . . . . . . . . . . . . . . . . . . . . . . .. | 2 |
| NO, not limited at all . . . . . . . . . . . . . . . . . . . . . . . . . . . . . . . . . | 3 |

1. How much **bodily** pain have you had during the **past 4 weeks**?

(Circle one number)

| None . . . . . . . . . . . . . . . . . . . . . . . . . . . . . . . . . . . . . ……... . . | 1 |
| --- | --- |
| Very mild . . . . . . . . . . . . . . . . . . . . . . . . . . . . . . . . . . . . . . . . . | 2 |
| Mild. . . . . . . . . . . . . . . . . . . . . . . . . . . . . . . . . . . . . . . . . . . . .. . | 3 |
| Moderate . . . . . . . . . . . . . . . . . . . . . . . . . . . . . . . . . . . . . . . . .. | 4 |
| Severe . . . . . . . . . . . . . . . . . . . . . . . . . . . . . . . . . . . . . . . . . . .. | 5 |
| Very severe. . . . . . . . . . . . . . . . . . . . . . . . . . . . . . . . . . . . . . . . | 6 |

1. During the **past 4 weeks**, how much did **pain** interfere with your normal work (including both work outside the home and housework)?

(Circle one number)

| Not at all . . . . . . . . . . . . . . . . . . . . . . . . . . . . . . . . . . . . . . . . . | 1 |
| --- | --- |
| A little bit . . . . . . . . . . . . . . . . .. . . . . . . . . . . . . . . . . . . . . . . . . | 2 |
| Moderately. . . . . . . . . . . . . . . . . . . . . . . . . . . . . . . . . . . . . . . . | 3 |
| Quite a bit . . . . . . . . . . . . . . . . . . . . . . . . . . . . . . . . . . . . . . . . . | 4 |
| Extremely . . . . . . . . .. . . . . . . . . . . . . . . . . . . . . . . . . . . . . . . . | 5 |

These questions are about how you feel and how things have been with you **during the past 4 weeks**. For each question, please give the one answer that comes closest to the way you have been feeling.

1. How much of the time during the **past 4 weeks** have you felt so down in the dumps that nothing could cheer you up?

(Circle one number)

| All of the time . . . . . . . . . . . . . . . . . . . . . . . . . . . . . . . . . . .. . . | 1 |
| --- | --- |
| Most of the time . . . . . . . . . . . . . . . . . . . . . . . . . . . . . . . . . . . . | 2 |
| A good bit of the time. . . . . . . . . . . . . . . . . . . . . . . . . . . . . . . . | 3 |
| Some of the time. . . . . . . . . . . . . . . . . . . . . . . . . . . . . . . . . . . . | 4 |
| A little of the time . . . . . . . . . . . . . . . . . . . . . . . . . . . . . . . . . . . | 5 |
| None of the time. . . . . . . . . . . . . . . . . . . . . . . . . . . . . . . . . . . . | 6 |

1. How much of the time during the **past 4 weeks** have you felt calm and peaceful?

(Circle one number)

| All of the time . . . . . . . . . . . . . . . . . . . . . . . . . . . . . . . . . . .. . . | 1 |
| --- | --- |
| Most of the time . . . . . . . . . . . . . . . . . . . . . . . . . . . . . . . . . . . . | 2 |
| Some of the time. . . . . . . . . . . . . . . . . . . . . . . . . . . . . . . . . . . . | 3 |
| A little of the time . . . . . . . . . . . . . . . . . . . . . . . . . . . . . . . . . . . | 4 |
| None of the time. . . . . . . . . . . . . . . . . . . . . . . . . . . . . . . . . . . . | 5 |

1. How much of the time during the **past 4 weeks** did you have a lot of energy?

(Circle one number)

| All of the time . . . . . . . . . . . . . . . . . . . . . . . . . . . . . . . . . . .. . . | 1 |
| --- | --- |
| Most of the time . . . . . . . . . . . . . . . . . . . . . . . . . . . . . . . . . . . . | 2 |
| Some of the time. . . . . . . . . . . . . . . . . . . . . . . . . . . . . . . . . . . . | 3 |
| A little of the time . . . . . . . . . . . . . . . . . . . . . . . . . . . . . . . . . . . | 4 |
| None of the time. . . . . . . . . . . . . . . . . . . . . . . . . . . . . . . . . . . . | 5 |

1. How much of the time during the **past 4 weeks** did you feel worn out?

(Circle one number)

| All of the time . . . . . . . . . . . . . . . . . . . . . . . . . . . . . . . . . . .. . . | 1 |
| --- | --- |
| Most of the time . . . . . . . . . . . . . . . . . . . . . . . . . . . . . . . . . . . . | 2 |
| Some of the time. . . . . . . . . . . . . . . . . . . . . . . . . . . . . . . . . . . . | 3 |
| A little of the time . . . . . . . . . . . . . . . . . . . . . . . . . . . . . . . . . . . | 4 |
| None of the time. . . . . . . . . . . . . . . . . . . . . . . . . . . . . . . . . . . . | 5 |

1. How much of the time during the **past 4 weeks** have you been a happy person?

(Circle one number)

| All of the time . . . . . . . . . . . . . . . . . . . . . . . . . . . . . . . . . . .. . . | 1 |
| --- | --- |
| Most of the time . . . . . . . . . . . . . . . . . . . . . . . . . . . . . . . . . . . . | 2 |
| Some of the time. . . . . . . . . . . . . . . . . . . . . . . . . . . . . . . . . . . . | 3 |
| A little of the time . . . . . . . . . . . . . . . . . . . . . . . . . . . . . . . . . . . | 4 |
| None of the time. . . . . . . . . . . . . . . . . . . . . . . . . . . . . . . . . . . . | 5 |

1. How much of the time during the **past 4 weeks** did you feel tired?

(Circle one number)

| All of the time . . . . . . . . . . . . . . . . . . . . . . . . . . . . . . . . . . .. | 1 |
| --- | --- |
| Most of the time . . . . . . . . . . . . . . . . . . . . . . . . . . . . . . . . . . .. | 2 |
| Some of the time. . . . . . . . . . . . . . . . . . . . . . . . . . . . . . . . . . . | 3 |
| A little of the time . . . . . . . . . . . . . . . . . . . . . . . . . . . . . . . . . . | 4 |
| None of the time. . . . . . . . . . . . . . . . . . . . . . . . . . . . . . . . . . .. | 5 |

1. During the **past 4 weeks**, how much of the time has your **physical health or emotional problems** interfered with your social activities (like visiting with friends, relatives, etc.)?

(Circle one number)

| All of the time . . . . . . . . . . . . . . . . . . . . . . . . . . . . . . . . . . .. | 1 |
| --- | --- |
| Most of the time . . . . . . . . . . . . . . . . . . . . . . . . . . . . . . . . . . .. | 2 |
| Some of the time. . . . . . . . . . . . . . . . . . . . . . . . . . . . . . . . . . . | 3 |
| A little of the time . . . . . . . . . . . . . . . . . . . . . . . . . . . . . . . . . . | 4 |
| None of the time. . . . . . . . . . . . . . . . . . . . . . . . . . . . . . . . . . .. | 5 |

How TRUE or FALSE is the following statement for you.

17. I am as healthy as anybody I know

(Circle one number)

| Definitely true. . . . . . . . . . . . . . .. . . . . . . . . . . . . . . . . . . . . . . | 1 |
| --- | --- |
| Mostly true. . . . . . . . . . . . . . . . . . . . . . . . . . . . . . . . . . . . . . . . | 2 |
| Mostly false. . . . . . . . . . . . . . . . . . . . . . . . . . . . . . . . . . . . . . . | 3 |
| Definitely false. . . . . . . . . . . . .. . . . . . . . . . . . . . . . . . . . . . . . . | 4 |

1. How much of the time during the **past 4 weeks** were you discouraged by your health problems?

(Circle one number)

| All of the time. . . . . . . . . . . . . . . . . . . . . . . . . . . . . . . . . . .. . . . | 1 |
| --- | --- |
| Most of the time . . . . . . . . . . . . . . . . . . . . . . . . . . . . . . . . . . . . | 2 |
| A good bit of the time. . . . . . . . . . . . . . . . . . . . . . . . . . . . . . . . | 3 |
| Some of the time. . . . . . . . . . . . . . . . . . . . . . . . . . . . . . . . . . . . | 4 |
| A little of the time . . . . . . . . . . . . . . . . . . . . . . . . . . . . . . . . . . . | 5 |
| None of the time. . . . . . . . . . . . . . . . . . . . . . . . . . . . . . . . . . . . | 6 |

1. How much of the time during the **past 4 weeks** were you frustrated about your health?

(Circle one number)

| All of the time. . . . . . . . . . . . . . . . . . . . . . . . . . . . . . . . . . .. . . . | 1 |
| --- | --- |
| Most of the time . . . . . . . . . . . . . . . . . . . . . . . . . . . . . . . . . . . . | 2 |
| A good bit of the time. . . . . . . . . . . . . . . . . . . . . . . . . . . . . . . . | 3 |
| Some of the time. . . . . . . . . . . . . . . . . . . . . . . . . . . . . . . . . . . . | 4 |
| A little of the time . . . . . . . . . . . . . . . . . . . . . . . . . . . . . . . . . . . | 5 |
| None of the time. . . . . . . . . . . . . . . . . . . . . . . . . . . . . . . . . . . . | 6 |

1. How much of the time during the **past 4 weeks** did you feel weighed down by your health problems?

(Circle one number)

| All of the time. . . . . . . . . . . . . . . . . . . . . . . . . . . . . . . . . . .. . . . | 1 |
| --- | --- |
| Most of the time . . . . . . . . . . . . . . . . . . . . . . . . . . . . . . . . . . . . | 2 |
| Some of the time. . . . . . . . . . . . . . . . . . . . . . . . . . . . . . . . . . . . | 3 |
| A little of the time . . . . . . . . . . . . . . . . . . . . . . . . . . . . . . . . . . . | 4 |
| None of the time. . . . . . . . . . . . . . . . . . . . . . . . . . . . . . . . . . . . | 5 |

1. How much of the time during the **past 4 weeks** have you had difficulty concentrating and thinking?

(Circle one number)

| All of the time. . . . . . . . . . . . . . . . . . . . . . . . . . . . . . . . . . .. . . | 1 |
| --- | --- |
| Most of the time . . . . . . . . . . . . . . . . . . . . . . . . . . . . . . . . . . . | 2 |
| Some of the time. . . . . . . . . . . . . . . . . . . . . . . . . . . . . . . . . . . | 3 |
| A little of the time . . . . . . . . . . . . . . . . . . . . . . . . . . . . . . . . . . | 4 |
| None of the time. . . . . . . . . . . . . . . . . . . . . . . . . . . . . . . . . . . | 5 |

1. How much of the time during the **past 4 weeks** did you have trouble keeping your attention on an activity for long?

(Circle one number)

| All of the time. . . . . . . . . . . . . . . . . . . . . . . . . . . . . . . . . . .. . . | 1 |
| --- | --- |
| Most of the time . . . . . . . . . . . . . . . . . . . . . . . . . . . . . . . . . . . | 2 |
| A good bit of the time. . . . . . . . . . . . . . . . . . . . . . . . . . . . . . . | 3 |
| Some of the time. . . . . . . . . . . . . . . . . . . . . . . . . . . . . . . . . . . | 4 |
| A little of the time . . . . . . . . . . . . . . . . . . . . . . . . . . . . . . . . . . | 5 |
| None of the time. . . . . . . . . . . . . . . . . . . . . . . . . . . . . . . . . . . | 6 |

1. How much of the time during the **past 4 weeks** have you had trouble with your memory?

(Circle one number)

| All of the time. . . . . . . . . . . . . . . . . . . . . . . . . . . . . . . . . . .. . . | 1 |
| --- | --- |
| Most of the time . . . . . . . . . . . . . . . . . . . . . . . . . . . . . . . . . . . | 2 |
| Some of the time. . . . . . . . . . . . . . . . . . . . . . . . . . . . . . . . . . . | 3 |
| A little of the time . . . . . . . . . . . . . . . . . . . . . . . . . . . . . . . . . . | 4 |
| None of the time. . . . . . . . . . . . . . . . . . . . . . . . . . . . . . . . . . . | 5 |

The next set of questions are about your sexual function and your satisfaction with your sexual function. Please answer as accurately as possible about your function during the last 4 weeks only.

1. **During the past 4 weeks**, how much of a problem was the lack of sexual interest?

(Circle one number)

| Not a problem. . . . . . . . . . . . . . . . . . . . . . . . . . . . . . . . . . . . . . | 1 |
| --- | --- |
| A little bit of a problem. . . . . . . . . . . . . . . . . . . . . . . . . .. . . . . . | 2 |
| Somewhat of a problem. . . . . . . . . . . . . . . . . . . . . . . . . . . . . .. | 3 |
| Very much a problem. . . . . . . . . . . . . . . . . . . . . . . . . . . . . . . . | 4 |

**During the past 4 weeks**, have you had an active sexual life?

- YES
- NO

If the answer is YES go to the next question.

If the answer is NO go to the question **number 28**.

How much of a problem was each of the following for you **during the past weeks**?

(Circle one number on each line)

| **MEN** | Not a problem | A little of a problem | Somewhat of a problem | Very much a problem |
| --- | --- | --- | --- | --- |
| 1. Difficulty getting or keeping an erection | 1 | 2 | 3 | 4 |
| 1. Difficulty having orgasm | 1 | 2 | 3 | 4 |
| 1. Ability to satisfy sexual partner | 1 | 2 | 3 | 4 |

(Circle one number on each line)

| **WOMEN** | Not a problem | A little of a problem | Somewhat of a problem | Very much a problem |
| --- | --- | --- | --- | --- |
| 1. Inadequate lubrification | 1 | 2 | 3 | 4 |
| 1. Difficulty having orgasm | 1 | 2 | 3 | 4 |
| 1. Ability to satisfy sexual partner | 1 | 2 | 3 | 4 |

1. During the **past 4 weeks**, how much did *pain* interfere with your enjoyment of life?

(Circle one number)

| Not at all. . . . . . . . . . . . . . . . . . . .. . . . . . . . . . . . . . . . . . . . . . . . | 1 |
| --- | --- |
| Slightly. . . . . . . . . . . . . . . . . . . . . . . . . . . .. . . . . . . . . . . . . . . . . | 2 |
| Moderately. . . . . . . . . . . . . . . . . . . . . . . . . . . . . . . . . . . . . . . . . . | 3 |
| Quite a bit. . . . . . . . . . . . . . . . . . . . . . . . . . . . . . . . . . . . . . . . . . | 4 |
| Extremely. . . . . . . . . . . . . . . . . . . . . . . . . . . . . . . . . . . . . . . . . . | 5 |

1. Overall, how would you rate your own quality-of-life?

(Circle one number on the scale below)


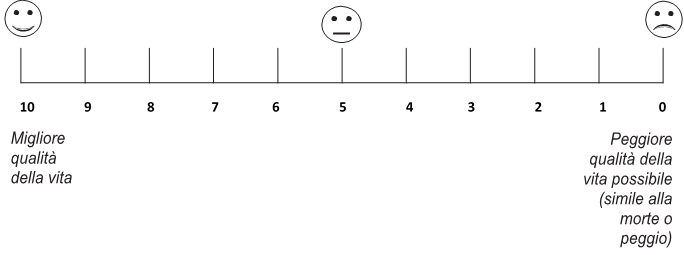


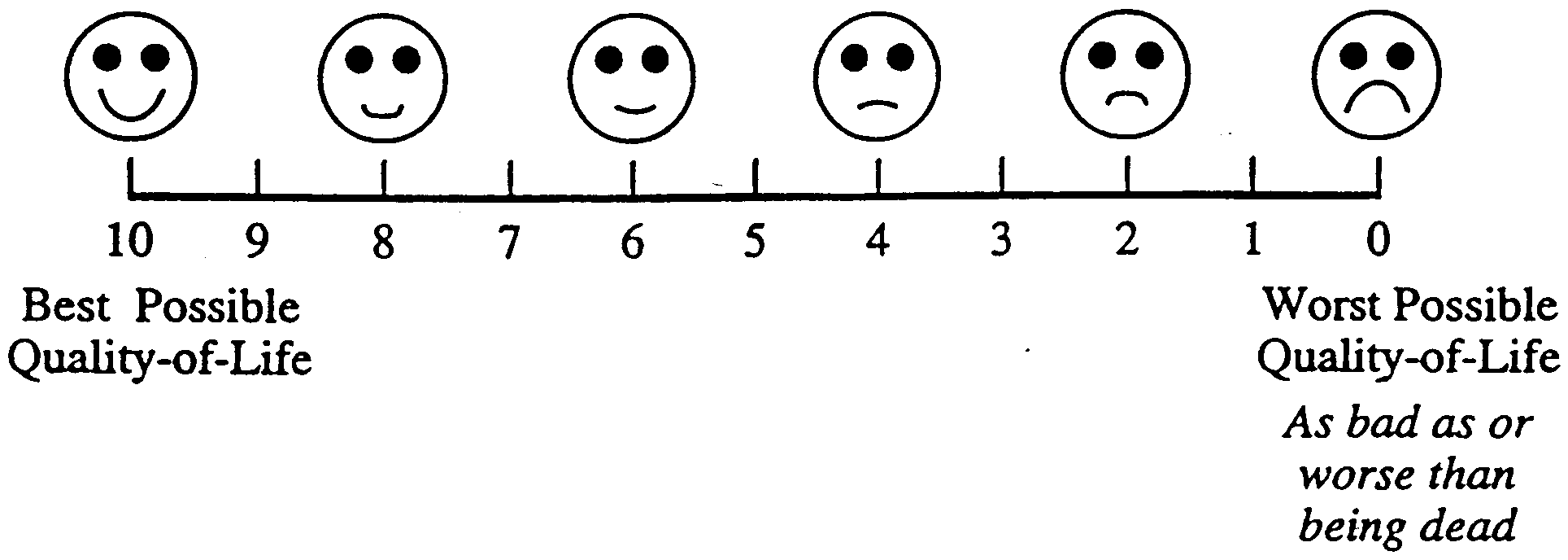

Supplement: S2 Appendix — (DOCX) [file pone.0153466.s002.docx]
