# Supplementary material for: Development of a Short Version of MSQOL-54 Using Factor Analysis and Item Response Theory
Source: PLoS One. 2016 Apr 14;11(4):e0153466. doi: 10.1371/journal.pone.0153466 (PMC4831784; doi:10.1371/journal.pone.0153466)
Supplement: S1 Fig — (PDF) [file pone.0153466.s004.pdf]

S1 Fig. Assumed [1] MSQOL-54 items-to-scales structure assessed for data fit by confirmatory factor analysis (CFA). Arrows indicate relationship indicated by CFA. Coefficients above each arrow are standardized regression weightings. Rectangles and ovals are observed and latent variables, respectively.

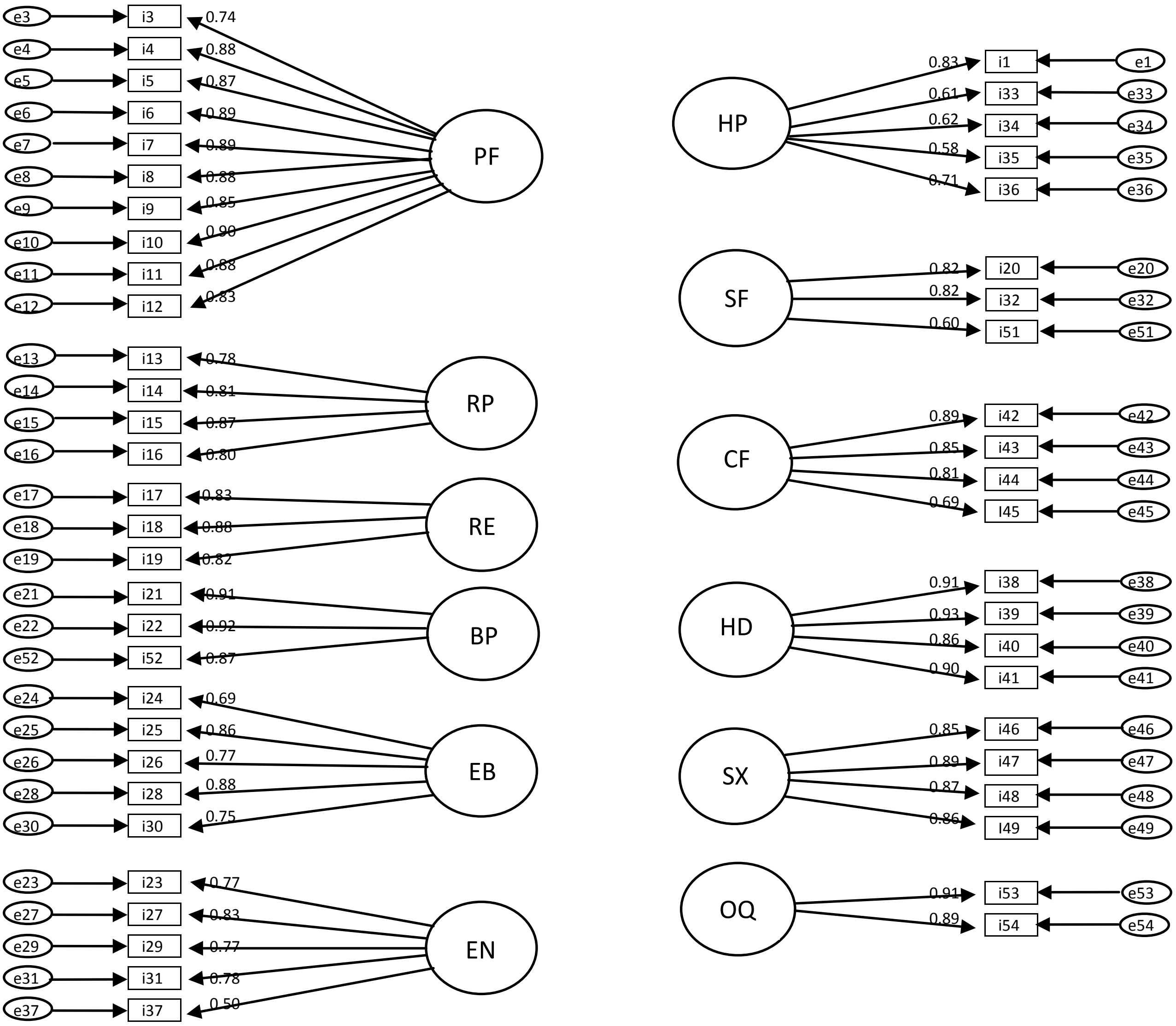

PF, Physical function; RP, Role limitations – physical; RE, Role limitations– emotional; BP, Bodily pain; EB, Emotional well-being; EN, Energy; HP, Health perceptions; SF, Social function; CF, Cognitive function; HD, Health distress; SX, Sexual function; OQ, Overall quality of life.

Correlations between all factors were significant (p<0.1) but are not shown in the Figure.

Model Fit

Fit statistic	Observed model fit	Criterion [2, 3]
Chi-square	2895.42	P>0.05
RMSEA	0.054	<0.08
CFI	0.98	≥0.95
SRMR	0.052	≤0.08

RMSEA, Root Mean Square Error of Approximation; CFI, Comparative Fit Index; SRMR, Standardized Root Mean Square Residual.

References

1. Vickrey BG, Hays RD, Harooni R, et al. A health-related quality of life measure for multiple sclerosis. Qual Life Res. 1995;4: 187–206.
2. Hu L, Bentler PM. Evaluating model fit. In Hoyle RH, ed. Structural equation modeling. Concepts, issues, and applications. London: Sage; 1995. pp.76–99.
3. Hu L, Bentler PM. Cutoff criteria for fit indexes in covariance structure analysis: Conventional criteria versus new alternatives. Structural Equation Modeling. 1999;6: 1–55.
