# Supplementary material for: Development of a Short Version of MSQOL-54 Using Factor Analysis and Item Response Theory
Source: PLoS One. 2016 Apr 14;11(4):e0153466. doi: 10.1371/journal.pone.0153466 (PMC4831784; doi:10.1371/journal.pone.0153466)
Supplement: S1 Table — (PDF) [file pone.0153466.s005.pdf]

S1 Table. Distribution of missing responses on MSQOL-54 Sexual Function and Satisfaction with Sexual Function subscales by gender.

**Item no.**	Men (N=209)	Women (N=426)	Total (N=635)
		No. (%)	
**Sexual Function**			
46	13 (6.2)	42 (9.9)	55 (8.7)
47	15 (7.2)	78 (18.3)	93 (14.7)
48	13 (6.2)	49 (11.5)	62 (9.8)
49	16 (7.7)	58 (13.6)	74 (11.7)
**Satisfaction with Sexual Function**			
50	15 (7.2)	54 (12.7)	69 (10.9)

MSQOL-54, Multiple Sclerosis Quality of Life-54.
