# Supplementary material for: Development of a Short Version of MSQOL-54 Using Factor Analysis and Item Response Theory
Source: PLoS One. 2016 Apr 14;11(4):e0153466. doi: 10.1371/journal.pone.0153466 (PMC4831784; doi:10.1371/journal.pone.0153466)
Supplement: S2 Table — (PDF) [file pone.0153466.s006.pdf]

S2 Table. Differential item functioning (DIF) of the MSQOL-29 by gender, age (40 years as cut-off), and EDSS score (2.0 as cut-off).

Item no.	Gender		Age		EDSS score	
	DIF	P value	DIF	P value	DIF	P value
**Physical function**						
9	0.03	0.91	0.18	0.51	0.18	0.53
4	0.09	0.77	0.40	0.14	0.43	0.17
6	0.09	0.75	0.24	0.41	0.00	1.00
5	0.43	0.13	0.21	0.44	0.46	0.13
7	0.13	0.68	0.03	0.92	0.42	0.25
11	0.35	0.27	0.24	0.44	0.26	0.56
**Bodily pain**						
21	0.25	0.24	0.03	0.88	0.04	0.87
22	0.03	0.91	0.15	0.51	0.08	0.75
52	0.40	0.12	0.12	0.22	0.04	0.86
**Emotional well-being**						
26[C]	0.30	0.13	0.66	<0.001	0.04	0.86
30[C]	0.00	1.00	0.09	0.64	0.47	0.02
25	0.21	0.19	0.47	0.001	0.30	0.07
**Energy**						
31[C]	0.05	0.78	0.02	0.90	0.23	0.23
27[C]	0.48	0.01	0.07	0.69	0.16	0.41
29[C]	0.43	0.02	0.00	1.00	0.05	0.78
**Cognitive function**						
44[C]	0.17	0.40	0.23	0.23	0.00	1.00
43	0.24	0.15	0.03	0.86	0.00	1.00
42[C]	0.19	0.35	0.29	0.13	0.00	1.00
**Health distress**						
38	0.08	0.68	0.11	0.53	0.18	0.36
41[C]	0.15	0.45	0.00	1.00	0.36	0.07
39	0.19	0.28	0.14	0.43	0.17	0.36
**Sexual function**						
46	0.40	0.07	0.30	0.16	0.04	0.86
47	0.36	0.09	0.11	0.59	0.13	0.58
48	0.37	0.09	0.06	0.79	0.15	0.53
49	0.37	0.08	0.12	0.56	0.03	0.90

EDSS, Expanded Disability Status Scale; MSQOL-29, Multiple Sclerosis Quality Of Life-29.
[C] Items with collapsed response categories
